# Chemically induced phenotype plasticity in the unicellular zygnematophyte, *Penium margaritaceum*

**DOI:** 10.1007/s00709-024-01962-x

**Published:** 2024-07-05

**Authors:** Josephine G. LoRicco, Kaylee Bagdan, Gabriel Sgambettera, Stuart Malone, Tawn Tomasi, Iris Lu, David S. Domozych

**Affiliations:** Department of Biology and Skidmore Microscopy Imaging Center, Skidmore College, 518 North Broadway, Saratoga Springs, NY 12866 USA

**Keywords:** Phenotypic plasticity, Zygnematophytes, Cytochalasin, Roscovitine, Extracellular matrix

## Abstract

Phenotypic plasticity allows a plant cell to alter its structure and function in response to external pressure. This adaptive phenomenon has also been important in the evolution of plants including the emergence of land plants from a streptophyte alga. *Penium margaritaceum* is a unicellular zygnematophyte (i.e., the group of streptophyte algae that is sister to land plants) that was employed in order to study phenotypic plasticity with a focus on the role of subcellular expansion centers and the cell wall in this process. Live cell fluorescence labeling, immunofluorescence labeling, transmission electron microscopy, and scanning electron microscopy showed significant subcellular changes and alterations to the cell wall. When treated with the actin-perturbing agent, cytochalasin E, cytokinesis is arrested and cells are transformed into pseudo-filaments made of up to eight or more cellular units. When treated with the cyclin-dependent kinase (CDK) inhibitor, roscovitine, cells converted to a unique phenotype with a narrow isthmus zone.

**Supplementary Information:**

The online version contains supplementary material available at 10.1007/s00709-024-01962-x.

## Introduction

Phenotypic plasticity is a common characteristic of many plants that defines their ability to respond to changes in the environment. Due to their sessile habit, plants have developed diverse strategies that alter their morphology, physiology, development, and reproduction in order to avoid, tolerate, and adapt to abiotic and biotic stressors (Schneider 2022; Pfennig 2021; Sultan 2000). This phenotypic plasticity includes major morphological changes in tissues and organs to more subtle modulations of cellular and biochemical components and their functions. Plant phenotypic plasticity has recently garnered significant interest primarily in response to escalating concerns about plant adaptations and survival due to climate change and the specific challenges therein (e.g., drought and high salt levels; Stotz et al. 2021).

The cell wall (CW) is a dynamic component of the plant extracellular matrix (ECM) that links the physical state of the cell with a variety of developmental and stress response mechanisms (Bacete and Hamman 2020). The CW and its specific components act as key environmental sensors that modulate phenotypic responses to stress, i.e., they are essential players in phenotypic plasticity. The plant cell constantly monitors the mechanochemical and functional integrity of the CW (cell wall integrity or CWI) and triggers signaling pathways in response to changes therein. For example, the CWI monitoring system perceives distortions of the CW-plasma membrane continuum, displacement of the plasma membrane, and external elicitors like CW fragments (Soni and Bacete 2023). These signals are transmitted to the cell that then regulates gene expression and subcellular programs that yield compensatory changes in CW structure and metabolism (i.e., a feedback loop between the living protoplast and the CW). These activities may lead to changes in cell/tissue size, shape, and physiology that more effectively cope with stress.

At the cellular level, phenotypic plasticity is a function of expansion and morphogenesis mechanisms. In plant cells, this requires an interplay between the structural architecture of the CW, the actions of the endomembrane and cytoskeletal networks, and internal turgor pressure (Cosgrove 2022). Likewise, these processes must coordinate with cytokinesis which leads to the production of a new CW between recently divided daughter cells. Division, expansion, and morphogenesis produce specific structural (e.g., shapes and sizes) and physiological features that conform the plant to its specific habitat and surrounding environmental stresses. Phenotypic plasticity also is believed to be a contributing factor in the evolution of various taxa (Leung et al. 2023; Gibbin et al. 2017; Fusco and Minelli 2010).

The streptophyte algae are the group of extant green algae that are most closely related and ancestral to land plants (McCourt et al. 2023). In the sister clade to land plants, the Zygnematophyceae, morphogenetic plasticity has been recognized even in taxa with simple morphology. For example, many species of *Spirogyra* consist of simple uniseriate filaments but upon wounding, form branched rhizoids (Ikegaya et al. 2008; Domozych and Bagdan 2022). Around 500+ million years ago (mya), an ancestral zygnematophyte successfully colonized land whose progeny ultimately gave rise to land plants (de Vries and Archibald 2018). (de Vries and Archibald 2018). These algae possessed the molecular toolbox that allowed them to survive and adapt to new terrestrial stresses such as high temperature, light, and CO_2_ as well as low O_2_ and major variations in the levels of water. Phenotypic plasticity very likely played a significant role in adapting to these new stresses yet very little is known about the processes involved therein. In this study, we employed the unicellular zygnematophyte, *Penium margaritaceum*, to initiate an analysis of morphological plasticity when placed under specific experimental stress conditions. The recent sequencing of the genome of *Penium* (Jiao et al 2020) has shown that this alga possesses expanded sets of gene families that regulate protective cellular features and physiological mechanisms associated with various stressors and that were likely involved in terrestrialization by ancient zygnematophytes. These include the biosynthesis, assembly, and remodeling of cell walls and signaling networks including the biosynthesis of some hormones/growth regulators and the biosynthesis of flavonoids. We describe the changes in cell expansion, morphogenesis, division, and CW architecture that occur when cells are interrogated with the pharmacological agents, cytochalasin E, and roscovitine.

## Materials and methods

### Cell culture

*Penium margaritaceum* was cultured in WHS medium (Woods Hole Medium) supplemented with 5% soil extract (Carolina Biological Supply) using previously described methods (Rydahl et al. 2015). Live cells from 7- to 10-day old cultures were harvested by centrifugation at 700 g for 1 min. The cell pellets were washed three times with fresh WHS and collected by centrifugation. Cells were added to 1 mL cultures of WHS containing an appropriate concentration of chemical agent in a 12-well uncoated cell culture plate (ThermoFisher Scientific). The wells were gently mixed, and the dish was sealed with Parafilm. Twelve-well cell culture plates were cultured at 22 °C under 74 µmol photons m^−2^ s^−1^ of cool white fluorescent light with a 16:8 h light–dark cycle for up to 1 week depending on the treatment.

Cytochalasin E (cyt E) and roscovitine were purchased from Sigma Chemical (St. Louis, MO, USA). Amiprophos-methyl (APM) and brefeldin A (BFA) were purchased from Sigma (USA). Cyt E treatment was performed at a concentration of 8 µg/mL (16.14 µM) for 3–6 days, and roscovitine treatment was performed at a concentration of 2 µM for 3–6 days. APM and BFA treatment concentrations were 1 µg/mL.

### Live cell labeling

#### Nuclear stain (Syto9)

Aliquots of treated cells were removed from the 12-well cell culture plate and added to 1.5-mL microcentrifuge tubes. After centrifugation at 4000 × g for 1 min, the supernatant was discarded from each tube, and the cell pellets were resuspended in fresh WHS. The tubes were vigorously shaken (to remove extracellular polymeric substances or EPS) and re-centrifuged. This represented the washing protocol for cells throughout the study. Washing was repeated twice more before labeling. Washed cell pellets were resuspended in 1 mL of deionized water or dH_2_O and incubated for 30 min in the dark with 2.5 µM Syto9 (Invitrogen). Cells were then washed 3 × with dH_2_O and imaged using a FITC filter set.

#### Reactive oxygen species (2,7 CFDA)

Washed cells were resuspended in WHS containing 5 µM 2,7 carboxyfluorescein diacetate (2,7 CFDA) and incubated for 30 min in the dark on a rotating platform. Cells were then washed 3 × with WHS and imaged with fluorescence microscopy using a FITC filter set.

#### Antibody labeling of CW

Washed cells were resuspended in WHS containing a 10:1 dilution primary antibody (JIM5, JIM7) in WHS. Cells were incubated for 90 min in the primary antibody and vortexed every 30 min. JIM5 incubation was performed in the dark on a rotating platform. JIM7 incubation was performed under 74 µmol photons m^−2^ s^−1^ of cool white fluorescent light on a rotating platform. Cells were then washed 3 × with WHS and resuspended in a 1:50 dilution of secondary antibody (Anti-Rat TRITC or Anti-Rat FITC; Sigma Chemical). Cells were incubated with the secondary antibody for 90 min, in the dark on a rotating platform. Cells were vortexed every 30 min. Cells were then washed 3 × with WHS and imaged using either the TRITC or FITC filter set.

To monitor cell wall expansion in recovering cells, JIM5 labeled cells were placed back into WHS (without chemical/enzymatic treatment) and placed back under the lights for 24–48 h. New growth was detected by the presence of unlabeled regions of CW.

### Fixed cell labeling

Control and treated cells were washed 3 × with WHS, and treated as follows:

#### Rhodamine phalloidin labeling

The cell pellets were resuspended in WHS + 0.1 mM MBS (m-maleimidobenzoyl-*N*-hydroxysuccinimide ester; Sigma Chemical) and incubated for 30 min in the dark. Cells were then washed 3 × with a Phalloidin Wash solution containing 50 mM PIPES, 5 mM MgCl_2_, 25 mM KCl, 5 mM CaCl_2_, and 5 mM EGTA (pH 6.9) and subsequently fixed in the fixative solution containing Wash + 1.9% formaldehyde (Sigma Chemical) solutions. The cells were washed 3 × in the Phalloidin Wash and then incubated in a solution of 1:500–1:250 dilution of rhodamine-phalloidin (Abcam) in Phalloidin Wash for 90 min in the dark. The cells were washed 3 × with Phalloidin Wash and imaged on an Olympus Fluoview 1200 confocal laser scanning microscope (CLSM).

#### Anti-tubulin labeling

Washed cells were incubated for 30 min in the dark in a solution of a microtubule wash consisting of 20 mM PIPES, 3.5 mM MgCl_2_, and 2 mM EGTA (pH 6.9) containing fixative 4% formaldehyde and 2.7% glutaraldehyde (Electron Microscopy Sciences, EMS; Fort Washington, PA, USA). The cells were then washed 3 × with the Microtubule Wash solution. Cells were then freeze-shattered using the protocol of Wasteneys et al. (1997). The shattered cells were then incubated for 30 min in phosphate-buffered saline or PBS containing 1% Triton-X. The cells were washed 3 × with PBS and then incubated for 30 min in PBS containing 13.2 mM NaBH_4_ (Sigma). The cells were washed 3 × with PBS and incubated for 10 min in PBS containing 10 mg/mL Driselase (Sigma). The cells were washed 3 × and incubated for 10 min in PBS containing 35 µM trypsin (Sigma). The cells were washed 3 × with PBS and then incubated for 10 min in PBS containing 50 mM glycine (Sigma). The cells were washed 3 × with PBS and incubated overnight in a solution of 1:800 Anti-Tyrosine Tubulin (Sigma T9028)/PBS. The cells were washed 3 × with PBS and then incubated for 90 min in a solution of 1:200 anti-mouse TRITC (Sigma)/PBS. The cells were washed 3 × with PBS. Imaged using CLSM with a TRITC or rhodamine filter set filter set.

### Transmission electron microscopy

#### Cryofixation

Washed cells were spray frozen using a commercial artist’s airbrush into 10 mL of liquid propane cooled to − 185 °C in liquid nitrogen. The frozen cells were poured into precooled (− 80 °C) glass scintillation vials containing 0.5% glutaraldehyde/0.2% tannic acid in acetone. The vials were placed in a − 80 °C freezer for 24 h. After 24 h, 0.1 g of osmium tetroxide was added to the scintillation vial, and the vial was placed back in the − 80 °C freezer for another 24 h. After this time, the vial was slowly warmed to room temperature over 16 h. The cells were then collected into a pellet by centrifugation at 700 × g for 1 min. The supernatant was discarded and the pellet was washed with acetone and recentrifuged. This was repeated twice more. The cells/pellet was then infiltrated for 3 h each in combinations of 25% Spurrs Low Viscosity Plastic (SLVP; EMS, USA)/75% acetone, 50% SLVP/50% acetone, and 75% SLVP/25% acetone at room temperature (RT). The cells were then placed in 100% SLVP for 2 h at RT. The cells were then pelleted into Beem capsules (EMS, USA) and polymerized at 55 °C for 8 h.

#### TEM imaging

For ultrastructural analysis, 70- to 100-nm sections were cut using a Diatome diamond knife with a LeicaUltracut microtome, collected on Formvar-coated copper grids (EMS, USA), and stained with UranyLess (EMS)/0/1% lead citrate and imaged on a Hitachi 7800 TEM at 120 kV.

#### Immunogold labeling

For immunogold labeling, 70-nm thick sections were collected on Formvar-coated nickel grids. The protocol of Domozych et al. (2014) was followed using JIM5 (Kerafast, USA) or CCRCM-80 (Complex Carbohydrate Research Center or CCRC, Georgia, USA). Controls included the omission of the primary antibodies.

## Results

**Fig. 1:**
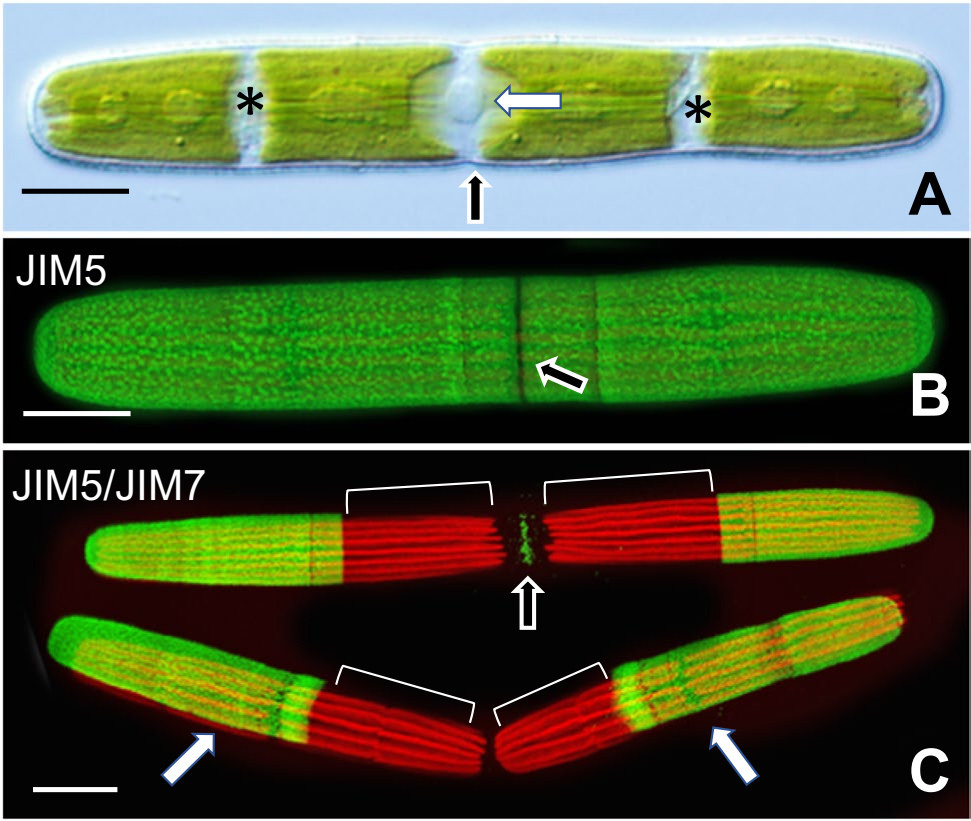
Penium phenotype and expansion/division dynamics. **A** The cylindrical cell consists of two semi-cells (*) surrounding the central isthmus (black arrow) that contains the nucleus (white arrow). **B** JIM5 labels the distinct homogalacturonan (HG) lattice of the outer cell wall (CW) layer. The narrow expansion zone (arrow) at the isthmus is unlabeled as the HG lattice has yet to form. **C** New HG deposited to the isthmus as labeled with JIM7 (black arrow). After the cell reaches a specific size, cytokinesis occurs, yielding two daughter cells (white arrows). Cells were labeled with JIM5 (green), placed back in culture, and then labeled with JIM7. New CW/cell growth in each cell is marked by brackets. Scale bars, 15 µm

*Penium margaritaceum* (Fig. 1a) forms distinct phenotypes when incubated in the actin-disrupting drug, cytochalasin E, and the cyclin-dependent kinase (CDK)-affecting agent, roscovitine. *Penium*’s typical phenotype is an elongated cylinder, with the cell center or isthmus surrounded by semi-cells that are equivalent in size. The cell wall (CW) is distinguished by an outer layer of homogalacturonan (HG) (Fig. 1b). New HG and other CW components are deposited in a narrow zone at the isthmus that subsequently push the older (“pre-existing CW) outward toward the poles of the cell (Fig. 1c). After the cell reaches a certain length, cell division occurs resulting in two daughter cells.

### Cytochalasin E treatment

**Fig. 2:**
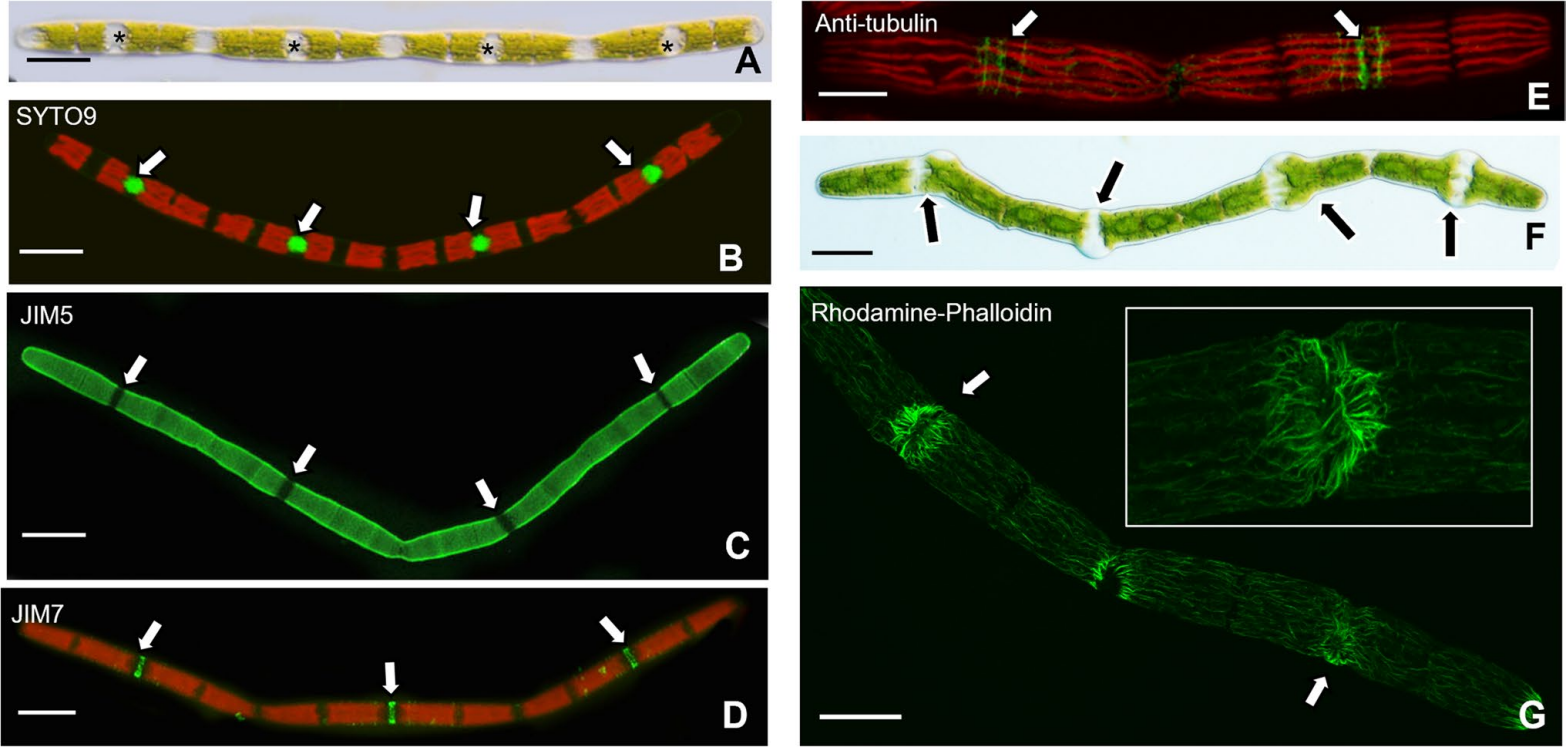
Effects of cytochalasin E (cyt E). **A** A pseudo-filamentous phenotype appears after 4 days of treatment. “Cell”-like units (*) make up the filament. Scale bar, 25 µm. **B** SYTO 9 labeling of pseudo-filament showing the position of a nucleus with each nucleus in the isthmus of a cell unit (arrows). Scale bar, 25 µm. **C** JIM5 labeling of the cell wall (CW) of a filamentous phenotype showing isthmus and CW synthesis zones of the cell units (arrows). Scale bar, 25 m. **D** JIM7 labeling of a filament showing the location of high esterified homogalacturonan (HG) secreted at the isthmus of each cell unit of the filament (arrows). Scale bar, 30 µm. **E** Labeling of microtubules with anti-tubulin antibody. Microtubule bands are found at the isthmus of each “unit” of the pseudo-filament (arrows). Scale bar, 20 µm. **F** The phenotype of the cell treated with cyt E for 4 days, washed and placed in 1 µg/mL amiprophos-methyl (APM) for 48 h. Note the swollen expansion zones (arrows). Scale bar, 15 µm. **G** Rhodamine-phalloidin labeling of cyt E-treated cell after 72-h treatment. Note the irregular aggregate of actin microfilaments in the isthmus (arrows, inset). Scale bar, 20 µm

When *Penium* is cultured in WHS medium containing 8 µg/mL (16.14 µM) cytochalasin E (cyt E), significant changes to the structural phenotype occur. Cyt E-treated cells transform into a filamentous-like phenotype (or pseudo-filament; Suppl. Movie S1). The pseudo-filament consists of multiple cell units, i.e., differentiated by zones where cell division did not occur. Each cell unit contains a nucleus (Fig. 2b) located within an expansion zone where new CW (e.g., HG) is deposited (Fig. 2c–d). These zones contain perpendicular bands of microtubules that are found in the isthmus and satellite zones of untreated cells (Fig. 2e; see also Ochs et al. 2014).

During treatment, cytoplasmic streaming was slower but was not arrested in the cortical region. Cells co-treated with cytE and the secretory inhibitor, brefeldin A (BFA) at 1 µg/mL for 4 days did not form pseudo-filaments and did not expand (Suppl. Figure 4a). When co-treated cells were washed and placed into the medium containing only cytE, the filamentous phenotype returns. In order to show that these zones are sites of expansion, some cyt E-treated cells that had formed pseudo-filaments (~ 4 days) were washed free of cyt E and placed in WHS containing 1 µM of the microtubule-disrupting agent, amiprophos-methyl or APM (Domozych et al. 2020). APM is thought to affect CW integrity at the expansion zone(s) resulting in a localized loss of tensile strength that yields to turgor pressure and causes swelling. In this study, APM treatment shows that cell expansion zones are indeed positioned where the nuclei, CW expansion zones, and microtubule bands are located (Fig. 2f).

In untreated cells, *Penium* contains parallel bands of microfilament bundles in the cortical cytoplasm that run parallel to the long axis of the cell (Ochs et al. 2014). Actin bundles are observed in cyt E-treated cells but localized, significant disruption of the actin cytoskeleton is observable (Fig. 2g, arrows/inset). These areas are positioned at or near the expansion zones of the individual cell unit.

**Fig. 3:**
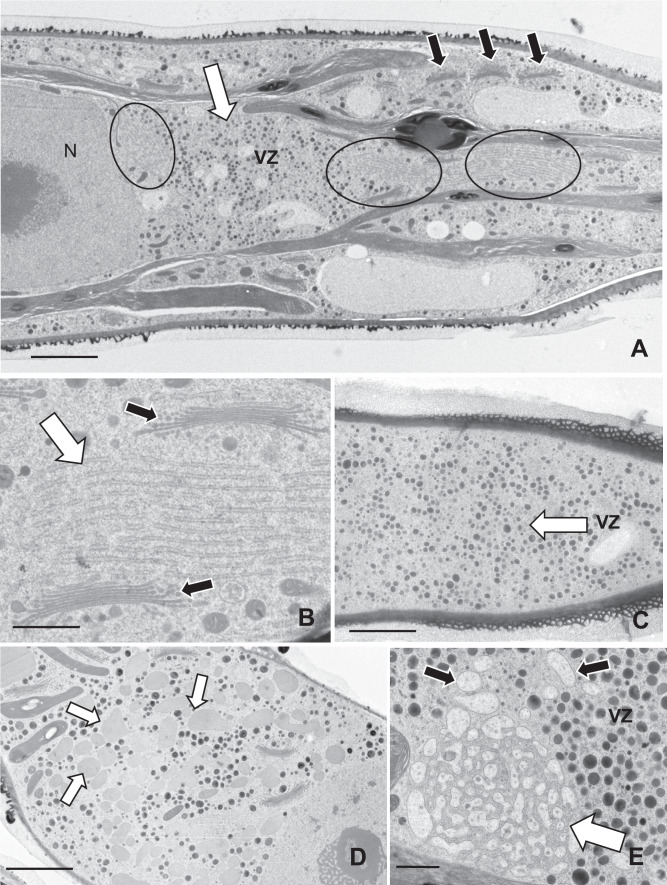
Ultrastructural changes in cyt E-treated cells. **A** Large vesicle-rich zones (VZ, white arrow) of cytoplasm appear in the isthmus region of treated cells. Aggregates of parallel-aligned sheets of ER (circles) are also found here interspersed with Golgi bodies (black arrows). Scale bar, 2 µm. **B** Magnified view of the parallel sheets of ER (white arrow) in the vesicle-rich zone. Note the adjoining Golgi bodies (black arrows). Scale bar, 750 nm. **C** Overview of the extent of the vesicle zone (VZ, arrow) in the cortical cytoplasm of treated cells. Scale bar, 3 µm. **D** After longer treatments (48 h), vacuole-like components with fine fibrils are located in the vesicle zone (arrows). Scale bar, 3 µm. **E** After 96 h of treatment, the vacuole-like components (black arrows) aggregate to form vacuolar clusters (white arrows). Scale bar, 100 nm

An ultrastructural analysis of the expansion zones in cyt E-treated cells revealed significant alterations in the endomembrane system architecture. In and around the expansion zones of cells treated for 3–4 days are cytoplasmic regions that are rich in vesicles (Fig. 3a and Suppl. Figure 1a) and contain multiple-layer aggregates of rough ER (Fig. 3a). A few Golgi bodies are found in this zone but most Golgi bodies are still positioned in typical linear arrays found in the valleys of cytoplasm created by the lobes of the chloroplast (Suppl. Figure 1b). Individual Golgi bodies retain the shape and *cis*–*trans* polarity as observed in untreated cells. The ER aggregates consist of 8–14 layers of ER tubes (Fig. 2b). The vesicle-rich cytoplasm can occupy large areas of the cell and contain an assortment of different types and sizes (Fig. 3c). After 5–6 days of treatment, the vesicle zone becomes filled with vacuoles that have light and homogenous osmiophilic lumens (Fig. 3d). After 7 days of treatments, the vacuoles aggregate into irregular masses and their contents contain tufts of fibrils (Fig. 3e).

**Fig. 4:**
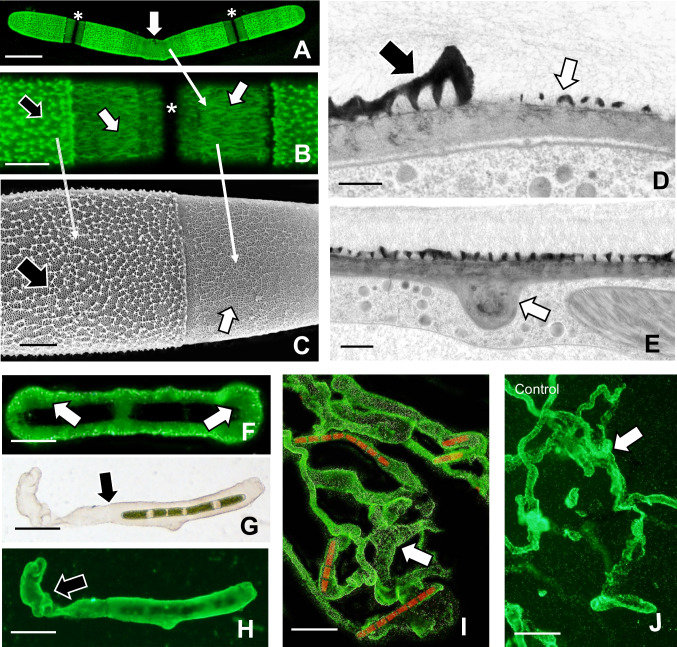
Effects of cyt E on the extracellular matrix (ECM). **A** JIM5 labeling of cyt E-treated cell for 48 h. While the homogalacturonan (HG) lattice on most of the cell remains intact, there is a subtle structural alteration at the isthmus (arrow). The two new expansion areas of the pseudo-filament are also visible (*). Scale bar, 20 µm. **B** Magnified view of the altered HG lattice of a treated cell after 48 h. The typical lattice (black arrow) on older parts of the cell surrounds the isthmus (*) containing a series of parallel fibers (white arrows). Scale bar, 8 µm. **C** Field emission scanning electron microscopy (FESEM) image of the interface of the pre-existing HG (black arrow) with the cyt E affected HG (white arrow). Note the difference in HG architecture. The corresponding zones in the confocal laser scanning microscopy (CLSM) images in A and B are highlighted by long arrows between images. Scale bar, 2.5 µm. **D** TEM interface between pre-existing CW (black arrow) and CW formed during cyt E treatment (white arrow). Scale bar, 400 nm. **E** TEM image of the ingrowth/septum (arrow) that sometimes forms in the isthmus area of treated cells. Scale bar, 500 nm. **F** Extracellular polymeric substance (EPS) secretion (arrows) around cell after 24-h treatment. The EPS ensheaths the cell. This cell was labeled with 0.75-µm fluorescent beads. Scale bar, 40 µm. **G** DIC image and **H** the corresponding fluorescence light microscopy (FLM) image of a cell beginning to glide after 48-h treatment. Note the EPS trail (arrow) at one end of the cell. Scale bars, 40 µm. **I** EPS trails in cells treated for 72 h (arrows). FLM image of fluorescent beads. Scale bar, 50 µm. **J** Control cells producing EPS in trails after 24 h (arrows). FLM image of fluorescent beads. Scale bar, 50 µm

We next examined the effects of cytE treatment on the ECM. JIM5-labeling of HG is still apparent on most of the cell surface (Fig. 4a), but structural changes to its architecture are found at the expansion zones (Fig. 4b). Field emission scanning electron microscopy (FESEM) imaging of the CW surface at an expansion zone shows distinct changes in the HG lattice formed during treatment versus that formed before treatment (Fig. 4c). The lattice projections of the regular CW are missing and are replaced by a covering of irregular fibers. TEM analysis of the interface zone between pre-existing CW and CW formed during treatment shows a significant reduction in the size of the lattice projections (Fig. 4d). The expansion zone of the cell units also often contained small ingrowths (Fig. 4e) from the inner CW layers.

We also monitored extracellular polymeric substance (EPS) secretion in treated cells. After 24-h treatment, the EPS trails formed during gliding in untreated cells (Fig. 4j) are not observed. Rather the cells secrete a thin layer of EPS that ensheaths the cell (Fig. 4f). However, after 48 h, the trails produced by secretion from one pole of the cell appear (Fig. 4g–h). After 72 h, long EPS trails are observed in treated cells (Fig. 4i).

### Roscovitine treatment

**Fig. 5:**
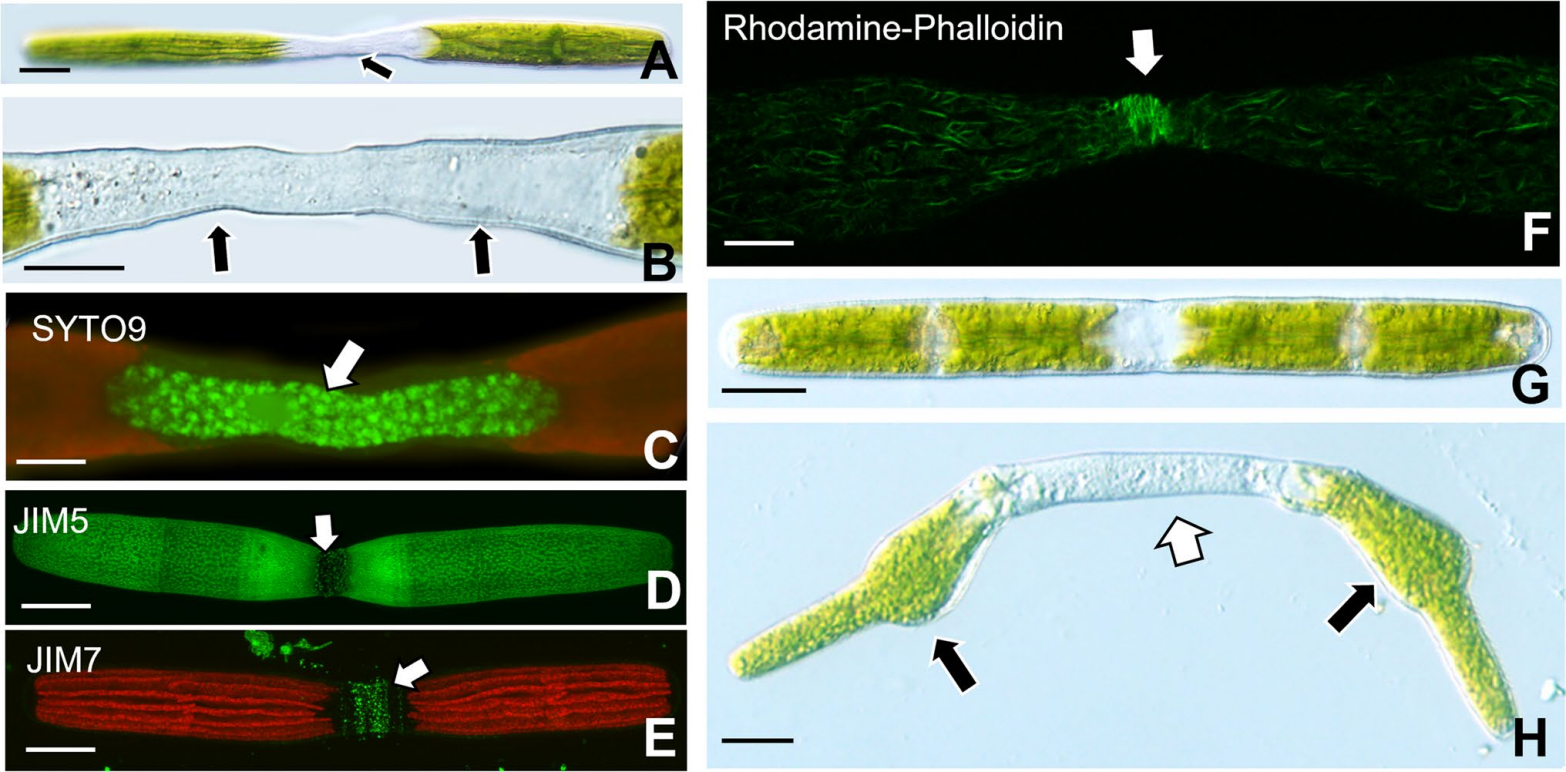
Effects of roscovitine. **A** When treated with roscovitine for 48 h, cells expand but narrow significantly at the isthmus (arrow). Scale bar, 15 µm. **B** Magnified view of cell treated for 72 h. Note that uneven morphology at the narrowing of the isthmus region (arrows). Scale bar, µm. **C** SYTO9 labeling of a cell treated for 48 h. Note the expanded nucleus (arrow) with multiple inclusive fluorescent entities. Scale bar, 8 µm. **D** JIM5 labeling of the homogalacturonan (HG) lattice. Note the interruption of labeling at the narrow isthmus (arrow). Scale bar, 12 µm. **E** JIM7 labeling of a cell treated for 48 h. Note that the label consists of scattered puncta (arrow) instead of a narrow band found in untreated cells (Domozych et al. 2020). Scale bar, 10 µm. **F** Rhodamine-phalloidin labeling of actin cables in cell treated for 48 h. Note the band in the isthmus (arrow). Scale bar, 10 µm. **G** When cells are co-treated with roscovitine and 1 µg/mL amiprophos-methyl (APM), no elongation is observed. Scale bar, 12 µm. **H** When cells are treated for 48 h with roscovitine, washed, and then treated with 1 µg/mL APM for 48 h, swellings (black arrows) occur at the satellite expansion centers. The central expansion of the cell is still apparent (white arrow). Scale bar, 15 µm

When treated with 2 µM roscovitine for 24 h or more, cells shrink in size at the isthmus (i.e., narrowing) to yield a “stretched out” phenotype (Fig. 5a). This isthmus zone contains the nucleus and cytoplasm with the chloroplasts displaced to the poles. The altered isthmus consists of irregular shapes (Fig. 5b). When labeled with the DNA label, Syto9, the nucleus appears notably elongated and filled with fluorescent puncta (Fig. 5c). Labeling of the HG lattice of the isthmus with JIM5 is much reduced (Fig. 5d) and JIM7 labeling is still located at the isthmus (Fig. 5e) but not in the narrow band that is seen in untreated cells (Domozych et al. 2020). Rhodamine-phalloidin labeling reveals both the cortical bundles of actin microfilaments and a dense cluster of microfilaments at the isthmus (Fig. 5f). When cells are treated with both roscovitine and 1 µM of secretory inhibitor brefeldin A (BFA; Domozych and LoRicco 2024), the changes to the isthmus do not occur (Fig. 5g, Suppl. Figure 4b). When BFA is removed and placed in roscovitine, the narrowing returns. Co-incubation of cells with 2 µM roscovitine and 1 µg/mL APM results in cells with the narrow isthmus phenotype, expected for roscovitine alone (Suppl. Figure 4c). When cells were incubated with roscovitine and APM sequentially (treated for 3 days in roscovitine, washed and incubated in 1 µg/mL APM) swellings are found at the satellite zones (Fig. 5h).

**Fig. 6:**
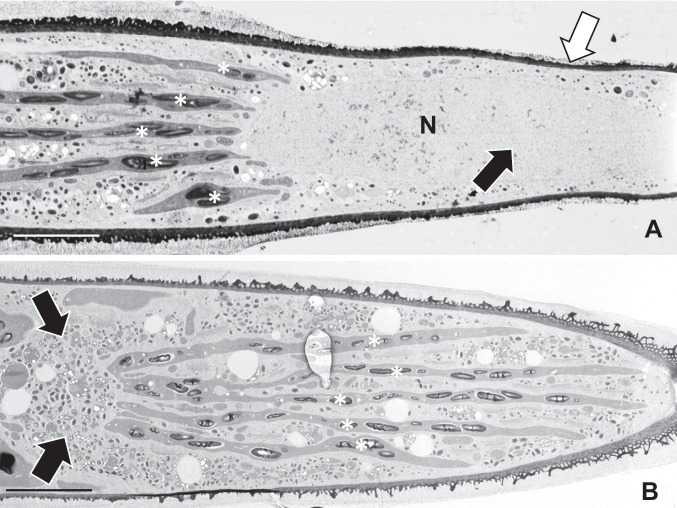
Ultrastructure of roscovitine-treated cells. Ultrastructure of roscovitine-treated cells. **A** The narrow isthmus zone (black arrow) of a cell treated for 72 h contains one expanded nucleus (N). At the polar regions, the chloroplast lobes (*) still define the narrow valleys of cytoplasm that contain ER and Golgi bodies. Scale bar, 4 µm. **B** Just outside the isthmus in a cell treated for 72 h exists an area of cytoplasm filled with vesicles and vacuoles (black arrows). At the polar regions, the chloroplast lobes (*) still define the narrow valleys of cytoplasm that contain ER and Golgi bodies. Scale bar, 4 µm

Ultrastructural examination of roscovitine-treated cells (4 days) shows that the narrowed isthmus zone contains the elongated nucleus (Fig. 6a). Just outside the nucleus is a cytoplasm containing large numbers of vesicles and some vacuoles (Fig. 6b). Changes to the CW are also apparent (see later). No aggregates of ER were found in these zones as had been observed in cyt E-treated cells. In the more polar regions of cells, valleys of cytoplasm surrounded by chloroplast lobes remained and contained Golgi bodies. Golgi body structure and positioning in the valleys appear unchanged when compared with untreated cells (Suppl. Figure 1c).

**Fig. 7:**
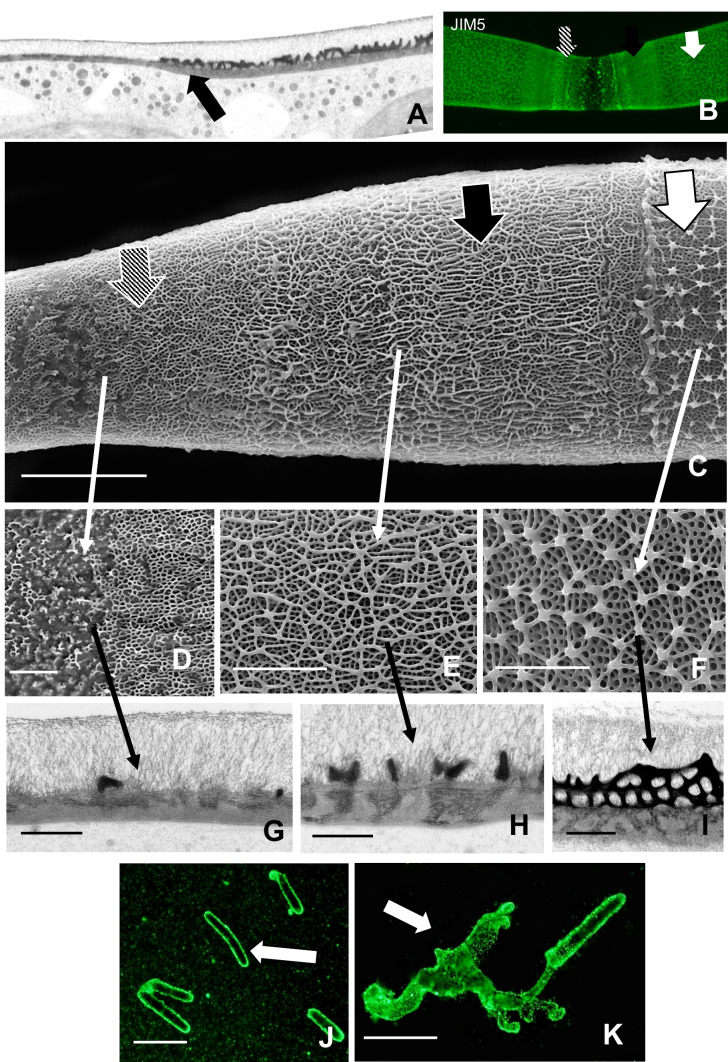
Changes to the extracellular matrix (ECM) upon treatment with roscovitine. **A** TEM image of the interface between the pre-existing CW (black arrow) and the CW formed during treatment for 72 h (white arrow). Note that the lattice is missing and the inner wall zone is thinner than that found in the pre-existing zone. Scale bar, 2 µm. **B** JIM5 labeled CW of the treated cell. Note the transition from the regular homogalacturonan (HG) lattice (white arrow) to the fibrillar transition zone (black arrow) and the disrupted lattice (striped arrow). Compare this live cell image with the electron micrographs below (**C–K**); 6 µm. Confocal laser scanning microscopy (CLSM) image. **C** SEM imaging of the CW surface of a cell treated for 72 h. Note the changes from the typical lattice of the pre-existing CW (white arrow), the transition zone where the HG forms irregular associations of fibers (black arrow) to the thin isthmus zone where the lattice is disrupted (striped arrow). Scale bar, 7 µm. **D**–**F** Close-up field emission scanning electron microscopy (FESEM) views of the individual zones of a treated cell as described in (**C**). Note the arrows identifying specific zones. **D** Scale bar, 3 µm; **E** scale bar, 200 nm; **F** scale bar, 150 nm. **G**–**I** TEM views of the individual zones of a treated cell as described in (**B**). **G** Scale bar, 500 nm; **H** scale bar, 500 nm; **I** scale bar, 400 nm. **J** EPS secretion after 24-h treatment. Note that extracellular polymeric substance (EPS) only ensheaths the cells (arrow). Scale bar, 60 µm. **K** EPS secretion after 72-h treatment. Note the production of EPS trails (arrow). Scale bar, 80 µm. **J** and **K** were incubated in fluorescent beads and then imaged with fluorescence light microscopy (FLM)

We next examined alterations to the CW within the narrowing isthmus. TEM imaging shows a loss of the HG lattice in the CW formed during treatment (Fig. 7a) that corresponds with the labeling observed with JIM5 labeling and CLSM imaging (Fig. 7b). FESEM imaging reveals a transition of structural changes to the HG layer of the CW (Fig. 7c). This transition goes from the regular HG lattice formed before treatment, a transition zone of HG fibers that do not form the typical projections of the lattice and a zone containing irregular masses of fibers (Fig. 7d–f). TEM imaging provides a more detailed imaging of the changes to the lattice (Figs. 7g–i).

EPS secretion is limited to a thin covering of the cell after 24-h treatment (Fig. 7j). However, after 24 h, EPS secretion produces the trails used in gliding (Fig. 7k).

### ROS labeling

Cells were labeled with 2,7 carboxyfluorescein diacetate (2,7 CFDA) to identify ROS-containing regions of the treated cells. No labeling was observed in control cells (Suppl. Figure 2a). Cyt E-treated cells are labeled at the isthmus zones of the cell units (Suppl. Figure 2b) and close-up imaging shows the labeling in the cytoplasm (Suppl. Figure 2c). The 2,7 CFDA labeling is found at the narrowed isthmus region of cells treated with roscovitine (Suppl. Figure 2d). Like that observed with cyt E, the labeling is observed in the cytoplasm (Suppl. Figure 2e).

### Recovery experiments

The effects of cyt E and roscovitine on the cell phenotype are reversible when cells are washed free of the agent and allowed to recover for several days in WHS. During the recovery of cyt E-treated cells, cell division most often occurred at active expansion zones, but fused regions between cell units did not recover. This led to the recovery of the normal phenotype for cells that divided off the ends of fused two-cell units (Suppl. Movie S2). Cells began division typically around 24 h and within a few days, pseudo-filaments were no longer observed in cultures. Roscovitine recovery followed two mechanisms. In cells that were treated for > 72 h, cell expansion and division occurred at the cell peripheries (Suppl. Figure 3b–c). Most likely the daughter nuclei moved to the satellite or peripheral expansion zones and cell division occurred here. In these cases, cells appear to be unable to divide at the narrow isthmus, and this zone is unable to recover (Suppl. Figure 3c–d). However, in cells treated for 48–72 h, cell division took place also at the original isthmus zone where the narrowing had occurred. Here, the daughter nuclei separate in the narrow isthmus region (Suppl. Figure 3a). A new cell plate (Suppl. Figure 3e–f) then formed yielding two daughter cells. However, the daughter cells had unusual phenotypes. The nuclei did not move to the new isthmus zones (Suppl. Figure 3 g–h) but remained in the polar zones of the daughter cells. Expansion occurred but yielded cells with one aberrant polar zone. This zone was stratified with a nucleus, Golgi zone, and vesicle zone (Suppl. Figure 3 h). These cells lived but did not divide further.

## Discussion

Cellular development in eukaryotes is centered around specific subcellular sites where the coordinated interplay between the endomembrane system, cytoskeletal network, plasma membrane, and extracellular matrix (ECM) leads to expansion, morphogenesis, and division. These sites are controlled by the activation of specific sets of genes during particular stages of the cell’s life and dynamically modulate in response to environmental stressors. In plant cells, development revolves around the cell wall (CW), a hydrated nanofibril-matrix composite that expands in response to chemical modulations and turgor pressure (Cosgrove 2022). This *plasticity* of the CW manifests in the formation of different sizes and shapes exhibited in the large diversity of plant cell types (i.e., phenotypic plasticity). These phenotypes are optimized for physiological functions such as photosynthesis, absorption of water and minerals, and interaction with external biotic and abiotic agents.

Zygnematophycean taxa are either unicellular or filamentous, have CWs notably similar in chemistry to that of land plant primary CWs (Sørensen et al. 2011), and whose development has been shown to employ specific subcellular centers of complexity (Pickett-Heaps 1975; Lütz-Meindl 2016; Domozych and Bagdan 2022). Yet, the structural organization and dynamics of these centers that lead to specific shapes including those that may have been important for the invasion of land remain to be discovered. In this study, we used the zygnematophyte, *Penium margaritaceum*, to investigate experimentally-induced phenotypic plasticity. The cell wall of *Penium margaritaceum* possesses notable similarities to that found in many land plants (Sørensen et al. 2011; Domozych and LoRicco 2023). Cellulose is the load-bearing component and is located in the inner layer of the cell wall. A distinct pectin domain highlights wall architecture and includes an outer lattice of calcium (Ca2 +)-complexed homogalacturonan and rhamnogalacturonan-I (Domozych et al. 2014). Arabinogalactan protein is found external to the lattice and functions as an adhesive agent (Palacio-López et al. 2019). *Penium*’s unicellular phenotype and well-organized subcellular architecture make it a valuable alga for understanding expansion, morphogenesis, and division in zygnematophytes (Davis et al. 2020; Domozych et al. 2014, 2020). *Penium*’s shape is simple, i.e., an elongated cylinder made of two “equal” semi-cells. Each semi-cell contains a multi-lobed chloroplast that defines valleys of cytoplasm that contain elongate mitochondria and ER along with linear arrays of Golgi bodies that produce various vesicle types that move to the cortical cytoplasm where they become part of an active, actin-mediated cytoplasmic streaming network. The morphogenetic center of the cell is the central zone between the chloroplast, the isthmus. The isthmus holds the nucleus and contains bands of microtubules and actin cables (Ochs et al. 2014). CW synthesis occurs at the isthmus, and the new wall displaces the older wall toward the poles. After attaining a specific length, the cell divides at the isthmus and cytokinesis entails a callose-containing septum (Davis et al. 2020).

### Cytochalasin E treatment and the formation of a “filamentous” phenotype

In order to investigate changes to cell shape we employed two different chemical stress agents, cytochalasin E (cyt E) and roscovitine. Cytochalasins are fungal-derived secondary metabolites that affect actin-based dynamics (e.g., perturb F-actin polymerization) and have also been shown to disturb gene regulation and signaling cascades, multiple types of membrane channels and transporters and phosphorylation patterns (Lambert et al. 2023; Holzinger 2022; Holzinger and Blaas 2016). Cyt E disrupts actin microfilament integrity and also perturbs the action of Na^+^ channels in membranes (Reifenberger et al. 2014). In the streptophyte alga, *Nitella pseudoflabellata*, cyt E treatment slowly arrested cytoplasmic streaming that resulted in the actual disruption of the subcortical actin bundle tracks on which myosin-dependent motility occurs (Foissner and Wasteneys 2007). In one cyt E treatment (i.e., lower concentration), the actin microfilament network remained but appeared as patches, swirling clusters, or short rods, similar to what was observed in cyt E-treated *Penium* labeled with rhodamine-phalloidin (compare Fig. 2f to Fig. 4a in Foissner and Wasteneys 2007). In *Penium*, cyt E treatment slows but does not arrest cytoplasmic streaming nor affect CW/cell expansion or post-mitotic movement of daughter nuclei to the expansion sites of the cell units of the pseudo-filament (i.e. the isthmus zones of the cell unit). When cells are co-treated with cyt E and BFA, cells do not expand or form pseudo-filaments. Once the BFA is removed and cells are placed in cyt E-containing medium, expansion and the formation of pseudo-filaments resume. BFA is an agent that perturbs Golgi dynamics, membrane trafficking, and secretion (Nebenführ et al. 2002; Robinson 2020). In a previous analysis of BFA effects on *Penium*, Golgi dynamics, secretion of ECM components and CW/cell expansion were stopped (LoRicco et al. 2023). In this study, BFA treatment again perturbs Golgi body function and stops the production of CW cargo-carrying vesicles needed for CW expansion. However, when the Golgi-based processing of CW components is allowed to recover and its CW-cargo vesicles travel to the cortical cytoplasmic streaming channels, cyt E treatment does not arrest their delivery to the expansion zones nor prevent CW expansion.

We also observed an expanded number of larger secretory vesicles (i.e., EPS-containing vesicles; Domozych et al. 2020) filling the cortical cytoplasm during cyt E treatment. We posit that cyt E treatment affects the secretion of EPS at least during the early stages of treatment. This corresponds with our observation of an initial arrest of EPS secretion (< 24 h) in cyt E-treated cells using fluorescent beads. After 24 h + treatment, EPS secretion resumed. This suggests that cells were able to adjust their secretory apparatus to offset the initial arrest of EPS secretion by cyt E treatment.

The effects noted above indicate that cyt E has different effects on secretion and membrane dynamics associated with the processing of two different ECM components, the CW and the EPS. Further studies will be required to determine if this phenomenon is due to different sets of actin microfilaments responsible for the secretion of the two ECM components. Microtubules have also been shown to participate in membrane trafficking in plants (Khoso et al. 2023), but little is known about their functions in zygnematophytes. Future studies will be needed to elucidate the microtubular network in secretion in *Penium*.

### Cytokinesis disruption by cytochalasin E

Cyt E treatment also arrests septum formation during cytokinesis that results in cell units attached end-to-end with each containing a central CW/cell expansion zone. Actin along with microtubules and associated proteins (e.g. myosins, kinesins) are fundamental components of plant cell division. They participate in defining the pre-division subcellular zones that predict the plane of cytokinesis (i.e., pre-prophase band or PPB), fuel mitosis, and regulate cell plate development during cytokinesis (Yuan et al. 2023; Sinclair et al. 2022, 2024, 2022; Jawaid et al. 2022). In the latter, these cytoskeletal components direct Golgi-derived vesicles carrying CW polymers to the cell plate and support its expansion and maturation leading to the formation of a new cross wall between daughter cells. Alteration of the actin network using pharmacological agents has been shown to affect the formation of the cytokinetic apparatus. In tobacco, BY-2 cells treated with latrunculin B, dispersed vesicles were observed residing at the equatorial plane of the cell plate instead of forming clusters that normally lead to the formation of the cell plate (Maeda and Higaki 2021; Maeda et al. 2020). In this study, the cytoplasmic zones located in cyt E-treated cells where cytokinesis should have occurred also contained large clusters of vesicles. It is very likely that cyt E, like other actin perturbation agents, also affected the formation of the cytokinetic apparatus by arresting vesicle fusion. Cytokinesis in *Penium* entails the formation of a callose-containing septum that grows inward from the side walls (Davis et al. 2020). The septum very likely entails a furrowing mechanism of the plasma membrane into the cell. Furrowing is a cytokinetic mechanism commonly found in many eukaryotes (Fraschini 2020; Pollard and O’Shaughnessy 2019), but the subcellular dynamics of this process in zygnematophytes remain unresolved. In other green algae like *Chlamydomonas*, furrowing requires phycoplast microtubules and very likely, actin but not type-II myosin (Cross and Umen 2015; Onishi et al. 2020).

*Penium*, like other streptophyte algae, does not produce a phycoplast during cytokinesis. It is likely that a distinct set of actin microfilaments (versus those involved in CW expansion and EPS secretion) is a necessary component for septum formation in *Penium*. Cytokinesis occurs rapidly in *Penium*, and capturing fine structural details remains a challenge. A furrow-derived septum containing callose has been described (Davis et al. 2020), but the existence of a cell plate/phragmoplast has not yet been identified. At present, it is also unknown if *Penium* also employs a phragmoplast/cell plate along with furrowing during cytokinesis like that found in the zygnematophyte, *Spirogyra* (Fowke and Pickett-Heaps 1969). Further studies are needed in order to dissect the different roles of actin during various cell developmental phases in *Penium.* This would also provide insight into evolutionary changes to subcellular components that were critical to the transition of the unicell to multicellular thalli in early divergent streptophytes.

In cells treated with cyt E for extended periods of time (4 to 5 + days), the dispersed vesicles of the cytokinetic zone appear to fuse together to form branched vacuoles that ultimately cluster together. Whether these structures represent altered vesicle fusion compartments formed during cyt E treatment or a type of autophagy/membrane retrieval apparatus remains to be elucidated. Stably transformed lines expressing fluorescent protein-cytoskeletal or endomembrane components are not yet available for *Penium* or any zygnematophyte, but their development will be of great value in dissecting membrane trafficking during development.

*Penium* contains a linear arrangement of Golgi bodies throughout the cell, and cyt E did not alter this arrangement, the architecture of the Golgi body, or the production of CW or EPS vesicles. However, cyt E treatment resulted in the formation of layers of ER near the large vesicle-rich zones of the cytoplasm. Golgi bodies were sometimes associated with these ER layers. In the more polar zones of treated cells, the ER-Golgi network typically found in untreated cells remains unchanged. ER and its arrangement in cells is dynamic and is often associated with actin in plants (Brandizzi 2021). These stacked layers of ER may be a manifestation of the need for enhanced CW component processing in the cytokinetic zone (i.e., the cell is effectively packing more ER membrane into more confined spaces and consequently enhances the ER’s biosynthetic machinery; Terasaki et al. 2013). Alternatively, the distinct zones made of layers of ER in cyt E-treated cells may be a feedback phenomenon whereby the inability of a septum to form from the vesicle network leads to a back-up of the ER-Golgi-vesicle mechanism that results in an organized hyperaccumulation of ER elements.

### Cytochalasin treatment and the CW

The homogalacturonan (HG) lattice is a distinct characteristic of the CW and has served as a marker for elucidating pectin dynamics in *Penium* (Domozych et al. 2021, 2014). We monitored lattice architecture in cells treated with cyt E and only one notable alteration was observed. While the HG fibers formed at the expansion zones of the cell units, they did not organize and produce the typical projections of the lattice. We propose that competent lattice formation is a function of precise quantities of HG secreted to a specific surface area of the underlying inner CW layers that are subsequently complexed with calcium (Ca^2+^). Cyt E treatment may alter this balance whereby the ratio of HG/underlying surface area is altered and the production of lattice projections is arrested.

### Roscovitine effects

In an ongoing pre-screening of over 30 different agents that target subcellular components and their functions, roscovitine treatment yielded a unique and reversible phenotype. We further investigated the effects of roscovitine (also known as Seliciclib and CYC202) in this study. Roscovitine (6-benzylamino-2-[1(*R*)-(hydroxymethyl) propyl amino-9-isopropylpurine) is an inhibitor of cyclin-dependent kinases (CDKs), which are important regulators of the plant cell cycle and transcription primarily during late G1 and G2 phases, and has also been shown to induce apoptosis in human cells (Aremu et al. 2012; Planchais et al. 1997). CDKs constitute a highly conserved group of serine/threonine kinases that form complexes with specific cyclins at different stages of the cell cycle and facilitate the phosphorylation of key target proteins necessary for advancing the cell cycle. In plants, A-, B-, and D-type cyclins have distinct roles in the cell cycle and dictate the timing of cell cycle transitions (Zheng 2022; Carneiro et al. 2021).

The major effect of roscovitine in *Penium* is a distinct narrowing of the cell at the isthmus. This phenotype is stable for at least 7 days and a return to a normal phenotype occurs during recovery via cell division and expansion. Additionally, roscovitine induces expansion of the nucleus and nuclear constituents, causes distinct changes to the cytoplasm at the isthmus, and results in the significant reduction of the homogalacturonan (HG) lattice.

The effects of roscovitine may be a result of endoreplication or reorganization of chromatin in the nucleus. Endoreplication is a process in plants whereby multiple rounds of DNA synthesis occur without cell division resulting in polyploid cells with increased DNA content (Shu et al. 2018). Roscovitine has been shown to induce this process (Soni and Bacete 2023). Endoreplication is regulated by a balance between CDK-cyclin complexes and CDK inhibitors that in turn influences turgor-driven cell expansion through the transcriptional control of cell wall (CW)-modifying genes (Bhosale et al. 2019). The narrowing of the cell and accompanying changes to CW architecture in the isthmus zone of *Penium* may be a manifestation of this reported effect. As the HG lattice is not the major load-bearing or shape-defining component of the CW (Domozych et al. 2021), the decrease in the HG lattice components at the narrow isthmus in roscovitine-treated cells indicates that the inner layers of the CW are the main affected zones. This unique “narrowing” effect along with different effects induced by other agents such as APM (e.g. swelling; Palacio-López et al. 2020) or cytochalasin E (pseudo-filament formation; see above) also demonstrates the value of a unicellular organism like *Penium* in investigating the subcellular bases of phenotypic plasticity. Additionally, the roscovitine effect may also involve the CW integrity (CWI) monitoring system of *Penium*. Plant cells can sense mechanochemical changes to their CWs that trigger signaling pathways and establish feedback loops between the protoplast and the CW (Soni and Bacete 2023; Vaahtera et al. 2019). The morphogenetic alteration caused by roscovitine may be in response to changes in the CW that trigger the CW monitoring system leading to the shape change.

### Cytoplasmic changes with roscovitine

During roscovitine treatment, the chloroplasts are displaced toward the cell poles leaving a large stretch of cytoplasm at the narrow isthmus. As the isthmus is the site of cell development and the focal point of significant changes due to chemical stress, movement of the plastids away from this zone may maintain photosynthesis efficiency under stress. This altered isthmus consists of the enlarged nucleus that is surrounded by cytoplasm with notably lower amounts of vesicles. In the zone between the central cytoplasm and the chloroplast are “pockets” of cytoplasm containing layers of ER, small mitochondria, and vacuoles. This segregation of organelles may be a means for enhancing their specific functions during stress (Terasaki et al. 2013). Likewise, this segregation may be a means of orderly aggregation of organelles for subsequent autophagy (Nozawa et al. 2009). Further work will be needed to determine this unusual organization of cytoplasmic components during chemical stress.

### Recovery at the “periphery” vs recovery at the isthmus

When cells treated for up to 10 days are washed free of cyt E and allowed to recover in a fresh growth medium, cell division occurs and produces cells with a normal phenotype. Cell division most often occurs following expansion within cell units, but “fused” zones between cell units do not appear to recover. Cyt E-induced effects begin at what would be the center zone of the pseudo-filament. There appears to be a point at which cytokinesis is irreversibly unable to occur at the central zone between the cell units, leading to permanently fused two-cell units. Division off the edges of a “fused” cell unit results in a normal cell phenotype, whereas the “fused” cells are unable to recover.

Recovery from cyt E treatment entails cell division focused at the periphery of fused cell units while recovery from roscovitine includes two mechanisms. (1) First, cell division may occur at the satellite expansion sites of the semi-cells. This must be preceded by recovery of the nucleus, mitosis, and transport of the daughter nuclei to the satellite zones. Expansion can be seen to occur at the satellite expansion zones following recovery from roscovitine by swelling induced by APM. Expansion at the satellite zones is then followed by division of cells at the periphery, while the disrupted (narrow) zone between the cell units does not recover, similar to the behavior seen in cyt E recovery. (2) Some cells, however, are able to divide at the narrow, altered isthmus indicating that the cell division can proceed here after removal of roscovitine. The daughter cells produced here are asymmetric, and one of their polar zones has highly contorted shapes. This may be due to initial expansion occurring at the contorted zones that possessed altered CW architecture that formed during treatment. After several cell division cycles, the normal phenotype of the daughter cells returns. These results show that cell division is the key process that rapidly restores order in the expression of the normal phenotype.

### ROS stress

2,7 carboxyfluorescein diacetate (2,7 CFDA) specifically labeled the zones where ROS is located and morphogenetic changes occur, i.e., the satellite expansion zones of the cell units of cyt E-treated cells and the narrow isthmus of roscovitine-treated cells. ROS is produced upon stress and signals diverse cascades of response mechanisms in plants (Tyagi et al. 2022; Mansoor et al. 2022). Our observations indicate that the chemical treatments used here induce oxidative stress that is localized in the expansion centers of the affected cells. ROS has previously been located in organelles of the zygnematophyte, *Micrasterias* (Darehshouri and Lütz-Meindl 2010; Lütz-Meindl, 2010), and future studies will be needed to elucidate its exact subcellular localization and subsequent molecular signaling in *Penium*.

## Conclusion

Phenotypic plasticity is a phenomenon in plants that adapts cells to specific developmental or stress-related phenomena. Using chemical agents that target different subcellular targets, we show distinct cellular and cell wall (CW) changes in *Penium*. Recent studies have posited that the evolutionary history of the Zygnematophyceae might represent a reductive evolution from a more complex ancestor of Zygnematophyceae and land plants (Hess et al. 2022; Donoghue and Clark 2024). Our demonstration of phenotypic plasticity especially with cytochalasin-treated cells suggests that changes to cytoskeletal-defined expansion and division centers may have been focal points for adaptations to stress leading to multicellularity. Future research will be enhanced once stable transformed lines expressing fluorescently-tagged proteins of the cytoskeletal and endomembrane systems are available for experimental work. Additionally, high-resolution imaging of the cytokinetic apparatus and the isthmus-based CW expansion zone that in turn, yield 3-dimensional models will shed valuable insight into cell development of zygnematophytes and the evolution of subcellular networks involved in multicellularity and the invasion of land.

## Supplementary Information

Below are the links to the electronic supplementary materials.

Supplementary figures S1-4 (PDF 634 kb)

Supplementary Movie S1 (AVI 70.5 MB)

Supplementary Movie S2 (AVI 45.7 MB)

## Data Availability

All data generated or analyzed during this study are included in this published article and its supplementary information files.
