# Supplementary material for: Chemically induced phenotype plasticity in the unicellular zygnematophyte, Penium margaritaceum
Source: Protoplasma. 2024 Jul 5;261(6):1233–49. doi: 10.1007/s00709-024-01962-x (PMC11511715; doi:10.1007/s00709-024-01962-x)
Supplement: Supplementary file 1 — Supplementary figures S1-4 (PDF 634 kb) [file 709_2024_1962_MOESM1_ESM.pdf]

Supplementary Information for:

Chemically induced phenotype plasticity in the unicellular zygnematophyte, *Penium margaritaceum*.

Josephine G. LoRicco[1*], Kaylee Bagdan[1], Gabriel Sgambettera[1], Stuart Malone[1], Tawn Tomasi[1], Iris Lu[1], David S. Domozych[1]

[1]Department of Biology and Skidmore Microscopy Imaging Center, Skidmore College, 518 North Broadway, Saratoga Springs, NY, 12866, USA.

[*]Corresponding author: Josephine LoRicco; jloricco@skidmore.edu

Suppl. Fig 1: The endomembrane components in treated cells

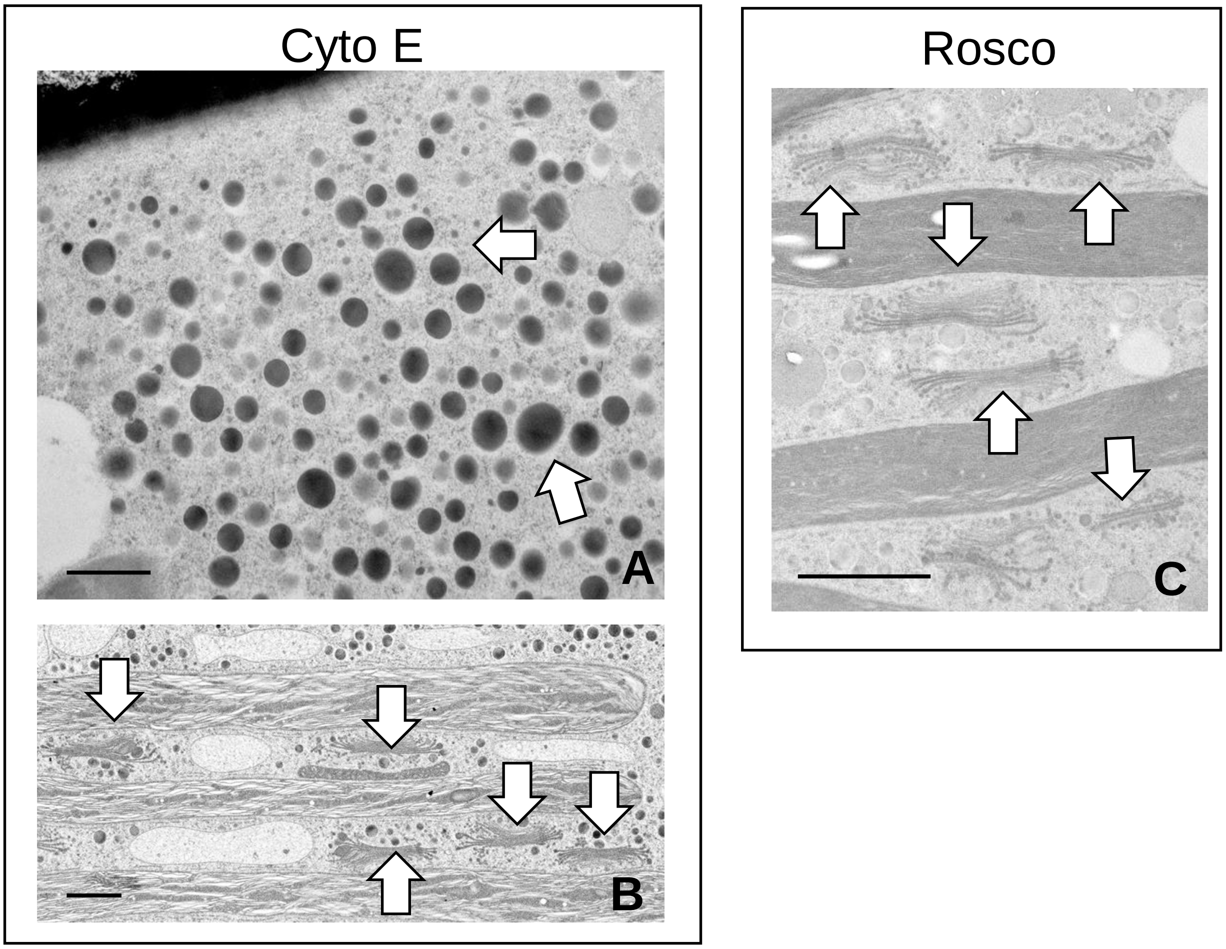

Suppl. Fig. 1 Endomembrane components in treated cells. (A) The peripheral cytoplasm of cyto E treated cells for 96+ h contain large numbers of secretory vesicles (arrows). Scale bar: 250 nm. (B) In cells treated with cyto E for 96+ h, the Golgi bodies (arrows). remain intact and positioned in the valleys of cytoplasm as seen in control cells. Scale bar: 500 nm. (C) The Golgi bodies (arrows) also remain intact and in the valleys of cytoplasm in roscovitine treated cells. Scale bar: 1 µm.

Suppl. Fig 2: 2,7 CFDA labeling

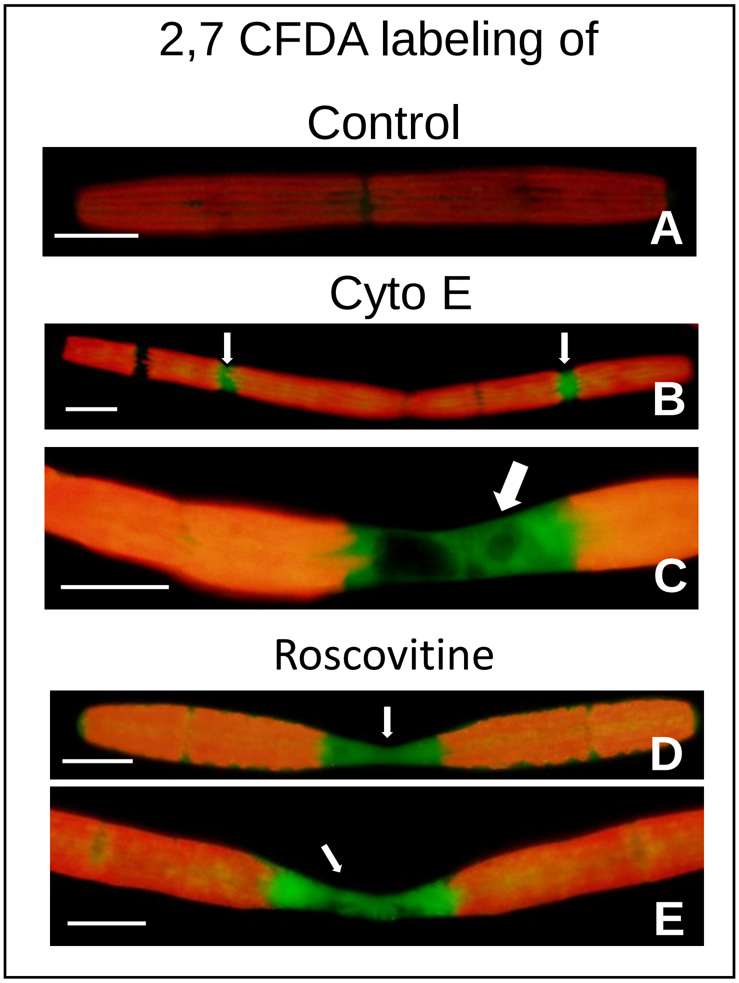

Suppl. Fig. 2 ROS localization and cytochalasin B treatment**.** (A) 2,7 CFDA labeling of control cell. No label is apparent.(B) 2,7 CFDA labeling of cell treated with cyt E for 48 b. Note the labeling (arrows) at the two isthmuses of the quasi-filament. (C) Magnified view of 2,7 CFDA labeling of a cell treated with cyt E for 96 h. Note the labeling of the cytoplasm (arrow). (D) 2,7 CFDA labeling of a cell treated for 48 h with roscovitine. Note the labeling of the expanded isthmus (arrow). (E) 2,7 CFDA labeling of a cell treated with roscovitine for 96 h. Note the labeling of the cytoplasm of the isthmus (arrow). All images were taken with FLM.

Suppl. Fig 3: Recovery of roscovitine treated cells

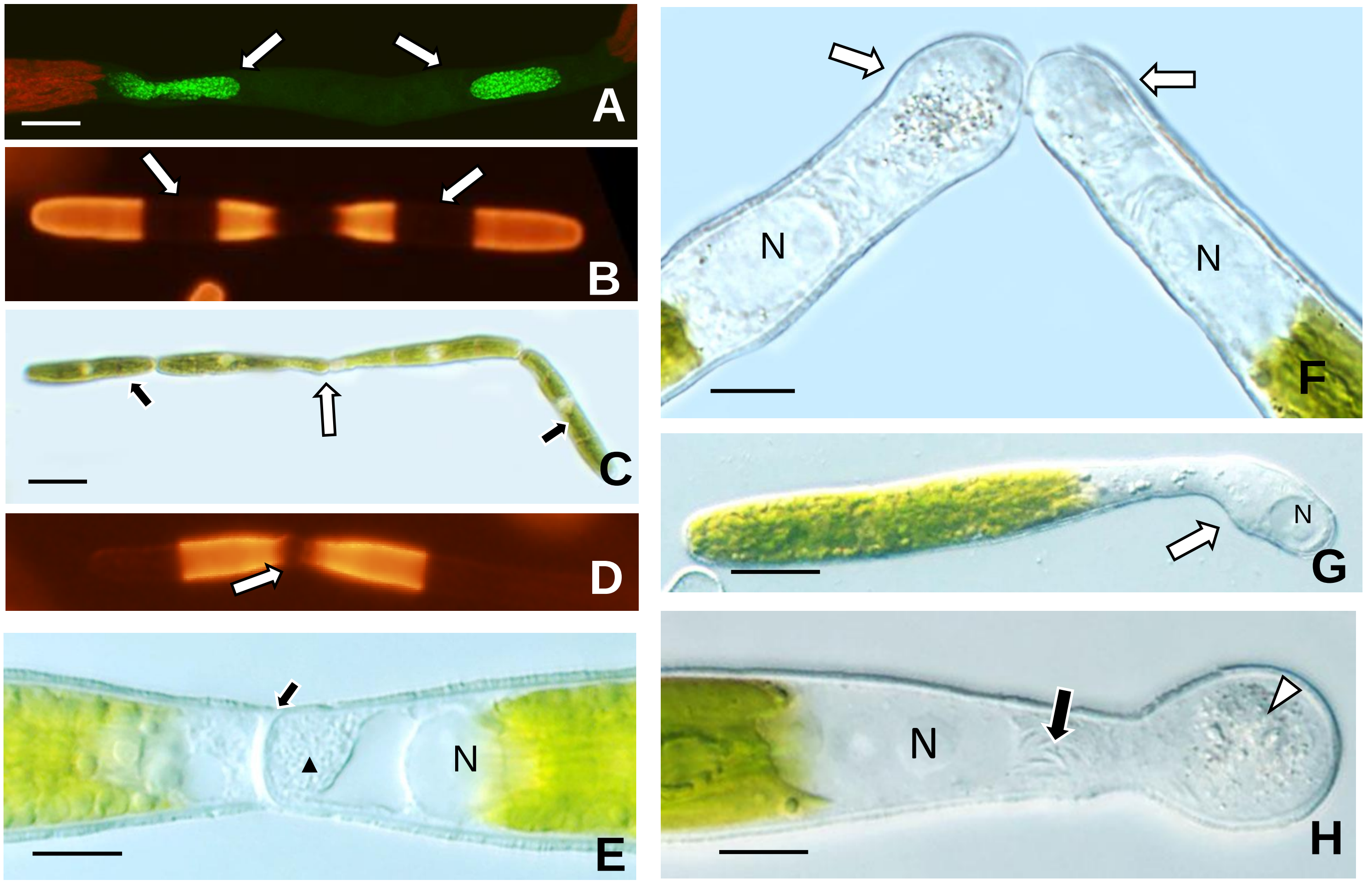

Suppl. Fig. 3 Recovery of roscovitine treated cells. (A) SYTO 9 labeled cell after 48 h recovery. Nuclei divide and separate toward the satellite expansion zones (arrows) Scale bar: 10 µm. CLSM image. (B) Roscovitine treated cell was labeled with JIM5, and allowed to recovery for 24h. Expansion can be seen to occur at the satellite expansion zones (arrows). FLM image (C) After 48 h recovery, cells may undergo cell division at satellite expansion zones that will yield daughter cells at the poles (black arrows). Scale bar: 20 µm. (D) Roscovitine treated cell was labeled with JIM5, and allowed to recover. No recovery of division is seen at the narrow isthmus zone after 72h (arrow). (E) In other cases, cell division may also occur at the original isthmus in some cells. Cytokinesis at the main or satellite isthmuses entails both a furrow and cell plate to form a new cross wall (black arrow). The cytoplasm at this zone is dense, i.e., filled with vesicles (arrowhead). Daughter nuclei (N) are found near the cytokinetic zone. Scale bar: 7 µm. (F) When cytokinesis occurs at the altered main isthmus, the daughter cells have large expanses of dense cytoplasm containing vesicles (arrows). Scale bar: 6 µm. (G) Daughter cell of cell where division occurred at the altered isthmus. Note the unusual shape (arrow) of the cell and the nucleus (N) is positioned at the poles . Scale bar: 15 µm. (H) Close up view of the unusual pole of the daughter cell formed as in €. Note the dense cytoplasm at the swollen pole (arrowhead), the collection of elliptical bodies (black arrow) and the nucleus (N). Scale bar: 7 µm. (D)-(H) are DIC images.

Suppl. Fig 4: Effects of co-treatments

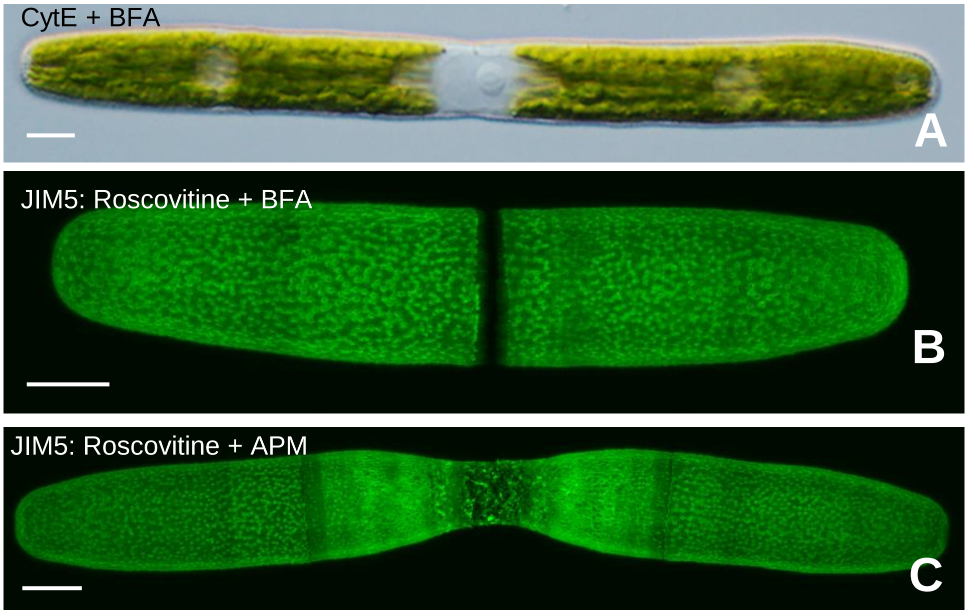

Suppl. Fig. 4 Effects co-treatments. (A) In *Penium* cells co-treated with CytE and BFA the cell did not expand and quasi-filaments did not form after 4 days. Scale bar is 10µm. (B) In JIM5 labeled *Penium* cells co-treated with roscovitine and BFA, the cell did not expand and no narrowing of the isthmus zone was seen after 4 days of treatment. Scale bar is 10µm. (C) Cells co-treated with roscovitine and APM for 3 days, result in cells with the narrow isthmus phenotype seen with roscovitine alone treatment. Scale bar is 10µm.

Legends for Supplementary Movies:

Movie S1: Formation of quasi-filaments of Penium. Timelapse video of *Penium margaritaceum* treated with 8 μg/mL cytochalasin E. Cells were embedded in 4% agarose and images were taken every 30 min on an Olympus Ix63 microscope using a 10x objective. Samples were illuminated the entire duration of the timelapse experiment with the transmitted lamp set to 6V. Cytokinesis was inhibited resulting in the formation of quasi-filaments of *Penium*.

Movie S2: Recovery of Penium from Cyt E treatment. *Penium margaritaceum* was treated with 8 μg/mL cytochalasin E for 3 days followed by labeling of the cell wall with the monoclonal antibody, JIM5. Cells were then washed and allowed to recovery in fresh WHS media while timelapse imaging was performed. Cells were embedded in 4% agarose and images were taken every 30 min on an Olympus Ix63 microscope using a 10x objective. Samples were illuminated the entire duration of the timelapse experiment with the transmitted lamp set to 6V. Cells were imaged both with brightfield and TRITC optics. In the brightfield channel (left) cells can be seen to divide – separating from the pseudo-filaments. In the TRTIC channel, regions of cell wall expansion can be seen a dark/unlabeled zones.
